# Okay, let's talk - short debriefings in the operating room

**DOI:** 10.1016/j.heliyon.2020.e04386

**Published:** 2020-07-04

**Authors:** Anna Sofie Mundt, Kirsten Gjeraa, Lene Spanager, Susanne Skovsø Petersen, Peter Dieckmann, Doris Østergaard

**Affiliations:** a Copenhagen Academy for Medical Education and Simulation (CAMES), Center for Human Resources and Education, Capital Region of Denmark, Denmark; b Department of Surgery, Hospital of North Sealand, Hilleroed, Denmark; c Department of Orthopedic Surgery, Hvidovre Hospital, Denmark; d Institute for Clinical Medicine, University of Copenhagen, Denmark; e Department of Quality and Health Technology, Faculty of Health Sciences, University of Stavanger, Norway

**Keywords:** Surgery, Abdominal surgery, Orthopedics, Educational development, Health profession, Medical education, Pedagogy, Cognitive psychology, Debriefing, Operating room, Learning, Teamwork, Non-technical skills

## Abstract

**Introduction:**

Debriefing is increasingly used to enhance learning and reflection in clinical practice. Nevertheless, barriers to implementing debriefings in the operating room (OR) include lack of time, the availability of trained facilitators, and difficulty gathering the full team after surgery. Spending five minutes on a debriefing during skin closure or between procedures may enhance learning and reflection on practice, generating to improve patient safety. The aim of this study was to explore characteristics, feasibility and content of short debriefings in the OR.

**Methods:**

This was a mixed-method study of short debriefings, analyzing audio-recordings, field notes and relevance ratings from multi-professional teams, that conducted short debriefings in the OR at two University Hospitals in Denmark.

**Results:**

A total of 135 debriefings were conducted, with a median duration of five minutes (range 1:19 min–12:05 min). A total of 477 team members participated in the debriefings. The teams’ median rating of relevance was 6 (range 1–10). The rating was higher following challenging events and in debriefings where the surgeon actively participated in the conversation. The teams discussed non-technical skills in all the debriefings and verbalized reflections on practice in 75 percent of the debriefings.

**Conclusion:**

It was feasible to conduct short debriefings in a production-focused, complex work environment. In all the debriefings, the teams discussed various non-technical skills (NTS) and reflected on practice. The majority of team members rated the debriefings as relevant for their task management.

## 1 Introduction

The operating room (OR) is a complex work setting, where team members’ non-technical skills (NTS) are crucial elements of the abilities required to perform safe and efficient patient treatments [1]. NTS are “cognitive, social and personal resource skills that complement technical skills, and contribute to safe and efficient task performance” [2]. NTS encompass “Situation Awareness”, “Decision Making”, “Communication and Teamwork”, and “Leadership” [3]. Addressing and developing these skills within the team and in relation to surgical procedures is a critical factor in avoiding adverse events [1].

NTS are often trained and discussed in facilitator-led debriefings in simulation-based courses either in simulation centers or *in situ* [4, 5]. However, demands for high levels of productivity and efficiency in the OR make it difficult to provide staff members time away from their assigned tasks for training and off-site training necessarily implies the need to apply what was learned in one setting in another setting. Therefore, identifying and taking advantage of daily clinical, inter-professional learning possibilities could be a potential strategy for creating relevant and necessary training opportunities that are less time demanding.

Feedback and debriefing are two of the methods utilized to enhance learning and reflection. In medical training, they are employed increasingly to enhance clinical performance [6]. For example, the National Health Service in England has added debriefings to their National Safety Standards for Invasive Procedures [7]. Although the majority of healthcare providers recognize the importance of debriefings, insufficient time, a lack of trained facilitators, and difficulties arranging settings for debriefings are cited as barriers to their implementation [8]. For the OR setting, one suggestion has been to use the time available at the end of a procedure, when the surgeon is closing up the skin, as an opportunity to debrief [9]. This could coincide with the ‘‘sign out’’ phase of the World Health Organization's Surgical Safety Checklist introduced in many countries [10]. The Surgical Safety Checklist has three phases to ensure safe surgery: sign in, time out, and sign out [10].

In the “sign out” phase (Which is conducted before the patient is leaving the OR) the team verbally confirms following: the name of the procedure recorded; that instrument, sponge and needle counts are correct; how the specimen is labelled; whether there are any equipment problems to be addressed and finally; the surgeon, anesthesia professional and nurse review the key concerns for recovery and management of this patient. Thus, the focus is on the management of the current patient's treatment. Therefore, this phase does not encourage the team to discuss or reflect upon any matters related to the team performance or patient safety issues that go beyond the immediate treatment of the current patient.

A tool known as TALK, developed by Diaz-Navarro and colleagues, has been developed to structure short clinical debriefings, lasting no longer than ten minutes [11]. A TALK conversation is designed to enhance learning and reflection on practice and can be led by any member of the team. TALK has four elements: T (Target): what should be discussed; A (Analyze): strategies used in the situation; L (Learning points): what can the team learn from the experience; and K (Key actions): what can be done to improve or maintain patient safety, and who will take responsibility for those actions [11]. The TALK tool encourages the team to discuss any clinical situation its members find important for improving patient care. This can include discussions about any relevant aspect for safety, quality, efficiency, etc. The TALK framework has similarities with other known debriefing structures and builds on literature supporting debriefing conducted in clinical and simulation setting [12, 13]. The TALK website provides more information (www.TALKdebrief.org). In order to understand the value of the tool, its concrete use in different contexts needs to be considered, in order to understand the dynamics, it triggers. Introducing debriefings in the OR could be a strategy for supporting reflection on practice; however, we do not know whether it is feasible in a production-oriented setting.

The benefits and disadvantages of different timings in relation to the procedure are not known. Learning from a debriefing is of little value to organizations unless it is transferred to clinical practice [14]. The insights gained by the teams from the debriefings and the intended actions expressed by the teams, as well as the value for the organization are not known.

The aim of this study was to explore the characteristics, feasibility and content of debriefings that were conducted based on the TALK tool in the OR. The unit of analysis is the TALK tool used in different context.

## 2 Materials and methods

This was an explorative mixed-method study using a deductive approach to analyze audio-recordings and written records with field notes from OR teams conducting debriefings to describe the use of the TALK tool in clinical debriefings. The study builds on the action research paradigm: the researchers are closely interacting with the unit of analysis, interventions, and descriptions of those, data collection, and analysis, iterate with each other.

### 2.1 Study design

Two OR departments at two University Hospitals in the Capital Region of Denmark participated in the study. Both departments were, based on convenience principles, invited to participate because they had previously been involved in projects regarding NTS and team training, leadership signaling a positive attitude toward NTS and debriefing. In close collaboration with the heads of the departments, the times and circumstances of the debriefings were determined. Taking their wishes into account was a condition to get field access. All clinicians at the departments were introduced to the project by the main author (ASM) at staff meetings and by e-mails, posters and flyers.

Each debriefing was led by a preselected facilitator to ensure completion and data collection. Each OR team, typically consisting of the scrub nurse, circulating staff, surgeon, surgical assistant, nurse anesthetist and anesthesiologist present, decided on the content of the debriefings by identifying situations during the procedures that they found relevant to discuss. The facilitators used the TALK tool to structure the debriefings and intended to commence the debriefings in a constructive, subject-oriented, manner. Debriefings should not compromise patient safety, e.g. by disturbing the ongoing work, so in collaboration with the OR team, the facilitator should determine the best timing. At both departments, all the facilitators (surgeons, anesthesiologists and OR nurses) attended a two-hours training course in facilitating TALK. Several staff members at both locations were experienced facilitators, having worked with simulation and debriefing for several years. Three of the twenty-two facilitators were also a part of the study team, of which one was the main author.

In Hospital A, the debriefings typically took place at the end of all orthopedic and general surgery procedures performed on patients under general anesthesia during the day over a four-week period (an estimated 160 possible debriefings). Here, the OR teams conducted the debriefings during skin closure. In Hospital A, none of the facilitators were employed in the department.

In Hospital B, all orthopedic procedures from three predetermined ORs with both alloplastic procedures and procedures on children were included to ensure variation in the teams attending the debriefings. The OR teams gathered five minutes before the arrival of the next patient to conduct the debriefings (an estimated 50 possible debriefings). Hospital B allocated ten days within a five-week period for the project. In Hospital B, all the facilitators were employed in the department.

### 2.2 Data collection

All debriefings were audio-recorded, and the facilitator completed a log after each debriefing that included demographic information about the team members, the duration of the debriefing, and a relevance score from each team member on a Likert-type scale ranging from 1 to 10 (1 = no relevance; 10 = highest relevance). Relevance was scored using the question: “How relevant did you find this debriefing for your task management?” Additionally, facilitators took field notes in this log.

### 2.3 Data analysis

First, all the audio-recordings were listened to by ASM. Sequences of the discussions were categorized with headings based on the citations and following, the NTS category was deductively coded into NTS elements by ASM. The coding system was created by combining the categories of the Danish tools to assess non-technical skills for surgeons NOTSSdk [15] and anesthesiologists’ non-technical skills ANTSdk [16], both adapted from the Scottish NOTSS tool for surgeons and the ANTS tool for anesthesiologists, respectively [3, 17]. NOTSS.dk comprise four overarching categories: “Situation Awareness”, “Decision Making”, “Communication and Teamwork”, and “Leadership”. Each category encompasses three to five elements that vary slightly between the tools. A few elements from ANTSdk were added to NOTSSdk e.g. the element “Planning and Preparing” was added to the Leadership category [16]. The content of the discussions could be coded into more than one NTS element since, typically, different angles are relevant.

Additionally, the audio-recordings were inductively coded for reflections on practice by marking the respective sequences in the recordings. These marked sequences could be described using two categories of verbalized reflections: a) “Gaining Insight”, defined as increased understanding of relationships between actions or events and their consequences, and b) “Intended Actions” for future practice, defined as explicitly stated intentions for future action. First, ASM listened to all the recordings. In a second coding step, ASM marked relevant sequences in the recordings as either “Gaining Insight” or “Intended Actions”. Double coding was possible. To ensure coding agreement, a second author (DO) listened to 20 percent of the audio-recordings and coded them separately. Field notes from the facilitators were used to deepen the understanding of feasibility and to capture reactions from the teams and the facilitator as well as any other relevant input. Relevance ratings were analyzed using T-tests for detecting differences between the hospital departments and between representatives of different professions attending debriefings.

Finally, the debriefings that the team members rated as highly relevant (team median ratings ≥9) or as of low relevance (team median ratings ≤2), were compared in terms of duration, team constellation, time of execution, themes discussed, dialogue characteristics, and reflections on practice.

The audio-recordings were analyzed using NVivo 11 Pro software (QSR International Pty Ltd).

### 2.4 Ethical considerations

Danish law exempts this type of research from Ethical Board approval, as no intervention in patient treatment takes place (J.-No.H-19002646). All staff members involved were informed about the study, that data would be anonymized and handled confidentially, and that they could withdraw at any point in time without further consequences. Debriefings were audio-recorded only when all team members in the OR had signed an informed consent form.

## 3 Results

### 3.1 Characteristics

In all, 135 debriefings were conducted. We excluded 31 debriefings due to poor sound quality in the audio-recording (n = 4), missing informed consent from team members (n = 13), lack of relevance ratings (n = 8), or when only two team members had participated (n = 8). Accordingly, in the following analysis, 104 debriefings were included. The median duration of the debriefings was 5:00 min (range 1:19 min–12:05 min). A total of 477 team members participated in the debriefing; see Table 1. All professions and specialties were represented in the study, several team members participated more than once and in Hospital A anesthesiologists managed to participate in 9 out of 104 debriefings (9%) whereas they managed to participate in 10 out of 14 debriefings (71%) in Hospital B.

**Table 1:** Team members included in debriefings in Hospital A and Hospital B, and duration of debriefings in minutes.

Hospital	Number of times a professional participated in a debriefing						Number of team members in all	Number of debriefings included	Duration in minutes Median (range)
	Scrub nurse	Circulating staff	Surgeon	Surgical Assistant	Anesthesia nurse	Anesthesiologist			
A	87	84	88	60	78	9	406	90	5:00 (1:19–12:05)
B	14	14	12	10	11	10	71	14	5:24 (3:23–8:00)
In total	101	98	100	70	89	19	477	104	5:00 (1:19–12:05)

The median relevance score of the debriefings was 6 (range 1–10); see Table 2. No significant differences were seen in the ratings between Hospital A and Hospital B.

**Table 2:** Relevance of debriefings, as rated by team members from 1 (not relevant) to 10 (most relevant).

Hospital	Relevance score Median (range)						
	Scrub nurse	Circulating staff	Surgeon	Surgical Assistant	Anesthesia nurse	Anesthesiologist	In all
A	7 (1–10)	6 (1–10)	7 (1–10)	5 (1–10)	6 (1–10)	6 (4–8)	6 (1–10)
B	7 (1–10)	6 (2–10)	5 (1–10)	3 (1–7)	6 (2–10)	5 (1–10)	6 (1–10)
T-test, p-value	0.58	0.95	0.45	0.12	0.41	0.26	0.46
Hospital A and B combined	7 (1–10)	6 (1–10)	7 (1–10)	5 (1–10)	6 (1–10)	5 (1–10)	6 (1–10)

### 3.2 Feasibility

In Hospital A, 114 of an estimated 160 possible (71%) debriefings were conducted; 90 of these were included in the study. In Hospital B, 21 of 50 (42%) estimated possible debriefings were conducted, and 14 were included. All in all, of the estimated 210 possible debriefings, 135 debriefings (64%) were conducted, and 104 debriefings were included.

In Hospital A, field notes revealed that teams did not have enough time to conduct debriefings at the end of short procedures. In addition, the teams argued that the debriefings took up too much time compared to surgical time and would interfere with safety procedures. Therefore, in agreement with the main author (ASM), some teams in the OR performing only short procedures, decided to conduct one debriefing at the end of the day. This has an impact on the number of possible debriefings and therefore for the response ratio. With our data set it is not possible to describe the actual size of this effect, but the reported response rate underestimates the actual response rate.

Field notes from Hospital A also described that team members who were too busy with tasks during the debriefings did not attend or did not contribute to the conversation. In addition, facilitators described that the teams were careful to adhere to the safety procedures, e.g. the first surgical count and not letting the debriefing interrupt. Moreover, several debriefings were terminated immediately if the condition of the patient changed or if the patient awoke.

Field notes from hospital B described difficulties for team members to gather for the debriefing due to lack of time, competing work tasks, misunderstandings regarding when the debriefing was taking place, and sudden logistic changes where the team would be separated and not be able to gather for a debriefing.

### 3.3 Content

#### 3.3.1 Non-technical skills

20 percent of the debriefings were coded by two raters. All discrepancies in coding found, could be resolved through discussion, and thereafter ASM did the coding only. In all the 104 debriefings, the teams discussed NTS. These skills were referred to 782 times in total. “Situation Awareness” and “Leadership” were the categories containing the most frequently coded elements; see Table 3.

**Table 3:** Non-technical skills utterances and reflections on practice, coded in debriefings and number of times each was coded (references).

Content	No. debriefings	No. references
**Non-technical skills utterances in debriefings**	**104**	**782**
• Situation Awareness	94	252
• Decision Making	23	55
• Communication & Teamwork	86	230
• Leadership	97	245
**Reflections on practice in all**	**78**	**179**
• Gaining Insight	66	108
• Intended Actions	54	71

“Situation Awareness”, represented by the element “Predicting and Thinking Ahead”, was mentioned in 69 of 94 debriefings. The scrub nurses’ abilities to predict and collect additional equipment before the surgeon needed it were emphasized as valuable. Sharing information that enabled other team members to react proactively was also highlighted, such as: “*Before I made the incision, she [the anesthesia nurse] told me it was bleeding a lot when she put in the IV line, so I started up very gently to prevent unnecessary bleeding. That was a really nice information to get*”. (Surgeon 113).

Leadership, represented by the elements “Planning and Preparing” and “Supporting Others”, was addressed in 85 of 97 debriefings. The team members identified good planning and preparing for the procedure, collecting essential equipment, and sharing relevant information about the patient, according to each situation, where everything “worked out fine”. Moreover, the importance of creating a positive atmosphere and establishing the appropriate professional tone was mentioned*: “I think it's amazing with the good atmosphere here, in the OR. Everything is said in a nice way with respect for each other. That means very much to me. I work much better when everybody speaks to each other in a proper way”.* (Scrub nurse 118).

“Communication & Teamwork”, represented by “Exchanging Information”, was discussed in 74 of 86 debriefings. Addressing or introducing colleagues by name when they enter the OR and using the WHO Surgical Safety Checklist for important information exchanges was appreciated. Clear commands and communication during procedures were additionally emphasized as important. Correspondingly, problems arising from a lack of communication was also described: “*We did not manage to inform the anesthesia staff. They did not know the OR was turned into a closed “flow” room”*. (Scrub nurse 103).

Decision Making, represented by “Considering Options”, was mentioned in 19 of 23 debriefings, e.g. failure to consider options and continuing to work despite inadequate conditions: “*The x-ray machine was not working properly, and we forgot to raise the operating table. That could have solved the problem [getting the x-ray machine into the right position to visualize the fracture]. But we were impatient to get on with the job”.* (Surgeon 16).

#### 3.3.2 Reflections on practice

Team members clearly expressed reflections on practice in 78 of the 104 debriefings; see Table 3.

“Gaining Insight” was the most frequently coded type of verbalized reflection. Insights related mostly to communication, as in the comment: “*It is nice when it is put into words what needs to be done*”. (Scrub nurse 56) Another comment on communication was: *“it is important to introduce yourself, especially when you are new*”. (Nurse anesthetist 58).

“Intended Actions” were explicit intentions to change future practices: *“Next time, when you call me because something is difficult, I won't leave before you tell me to”* (Surgeon 74); and *“I can see, that we would really benefit from waiting to receive a child in the OR until everybody is ready; you, me, anesthesia. I will ask our leaders about their attitude towards that. We have to hear what the people in charge have to say about that”*. (Surgeon 102).

Sometimes, “Intended Actions” were verbalized but immediately abandoned due to distrust in the management's ability or willingness to make changes: *“It is a very good idea to gather and brief each other before the next patient arrives in the OR, and it probably would solve this problem we've just had, but it is no use to bring it any further. No one will accept that anyway”.* (Surgeon 17).

#### 3.3.3 Description of highest- and lowest-rated debriefings

We looked at the highest-rated debriefings, with a median relevance rating above 9 (for 13 debriefings) and those with the lowest scores, with a median relevance rating of below 2 (for six debriefings). The 13 highest-rated debriefings had a mean duration of 6:09 min (range 03:27–12:05), and the six lowest-rated debriefings had a mean duration of 4:58 min (range 3:30–6:33). Team compositions were comparable in the highest- and lowest-rated debriefings, in terms of professions, disciplines, and number of participants involved. In both groups, the timing of the debriefings was equally distributed between conduction skin closure and gathering five minutes before the next patient arrived in the OR.

“Situation Awareness” and “Leadership” were categories where elements were particularly discussed in all the highest-rated debriefings; see the examples in Table 4. In the lowest-rated debriefings, no specific NTS element was discussed more than others. Regarding reflections on practice, the highest-rated debriefings had relatively more “Intended Actions” (21 sequences in 13 cases) compared to the lowest-rated debriefings (one sequence in six cases).

**Table 4:** Examples of non-technical skills utterances and reflections on practice discussed in the highest-rated debriefing (median 9 or 10).

Examples of non-technical skills discussed	Examples of reflections on practice
Situation Awareness	Gaining an insight
• High level of noise and talk in the OR while positioning patient during procedure caused tensions among team members. • High arousal and joking when converting from laparoscopic to open procedure due to bleeding stressed scrub nurse.	• It is important to maintain eye contact and communication, even when things are happening quickly. • Respect each other. • The importance of communication is emphasized. • The solution lies in gathering the team preoperatively to share information. • Be clear about who does what according to safety check lists. • Share information on work status. Let everyone know when you are ready to receive a new patient. • Use a closed loop technique when bringing information to the team. • Everyone is allowed to speak up.
Decision Making	
• Possibilities for performing the procedure using local analgesia; surgeon and anesthetist are praised for discussing the matter outside the OR and not within the uneasy patient's hearing range. • Decision not to follow standard procedures regarding positioning of patient leading to repositioning of patient during procedure.	
Communication & Teamwork	Indented actions
• Imprecise information shared regarding patient's conditions during procedure stressed the surgeon. • Information from “check in” procedure was not passed on to new team members, which resulted in unnecessary disturbances for the surgeon.	• Surgeon contacts resident surgeons about proper pre-op registration. • Nurse anesthetist defines list of important questions to ask before inducing patient. • Scrub nurse will read not only the procedure description but also the patient's file. • Nurse anesthetist will look into already-existing guidelines regarding “check in“ for children. • Surgeon will bring problematic issues regarding list of patients to the head of the department. • Scrub nurse will establish clear guidelines for handling phone calls and other interruptions. • Scrub nurse will support colleague to call for help if he/she is in distress.
Leadership	
• Useful information from the surgeon regarding planning made the team feel comfortable and confident. • Anticoagulants not adjusted pre-operatively, resulted in increased bleeding during the operation.	

As the field notes show, the dialogue in the highest-rated debriefings was characterized by members speaking to each other in a direct manner, addressing each other by first name, and using the pronouns “you”, “we” and “I”. The conversation was typically lively, and the surgeon played an active role in it. The teams mostly addressed problematic events; see the vignette in Table 5.

**Table 5:** Description of a highly rated debriefing.

Debriefing minutes: 7:22
The anesthesiologist and scrub nurses are gathered before a child is due to arrive in the OR. They discuss the planned procedure and consider the best way to sedate the patient. However, they are not fully aware of what the procedure implies. In the patient record, the procedure is estimated to last 90 min. On this basis, the child is anaesthetized. The surgeon has marked the side of the patient for surgery in the ward and arrives in the OR after the child has been anaesthetized. At the start of surgery, the two surgeons discuss the options and decide on a smaller, shorter procedure lasting only 15 min. The team decides to discuss about this in the debriefing.
During the debriefing, a team member proposes the possibility of the surgeon and anesthesiologist talking with each other before the child arrives. The surgeon recognizes the difficulties related to not being present during the “sign in” procedure in the OR but argues that procedures can change “on the fly” for the good of the patient, and this cannot always be predicted. There was a small possibility of a less-complicated procedure.
The anesthesiologist explains that he anaesthetized the child for a procedure that was expected to last 90 min and that he would have made another choice had he known that there was uncertainty about the duration of the procedure. He points out, that the solution to issues like this is to join the team in the OR before the child is anaesthetized.
At the end of the debriefing, another team member recalls that there is actually a guideline noting that the surgeon is obliged to participate in the “sign in” with very small children. The surgeon will pass this information on to everyone in the ward.

In the lowest-rated debriefings, we noted that individual team members spoke mostly to the facilitator, and the conversations were often quieter. In these debriefings, the teams mainly mentioned uneventful situations and good behavior among the team members.

Furthermore, in debriefings conducted during skin closure, more utterances such as “uhhh” and “what?” and “say that again” appeared, regardless of ratings. These were interpreted as signs of cognitive burden and communicative attempts to prevent overload.

## 4 Discussion

TALK debriefings were introduced for teams in the OR at two different hospitals. The conditions differed between the hospitals regarding: point of time of the debriefing, experience and position of the facilitators, and team composition. These differences might have an impact on the number of TALK debriefings conducted. This study emphasizes the need to study a tool like TALK in the organizational context in which it is used. In this discussion, we pinpoint differences and challenges at both sites and investigate them for effects. In all debriefings, the teams' discussed non-technical skills (NTS) and most frequently “Situation Awareness” and “Leadership” were mentioned. Reflections on practice were formulated by the team in 75% of the debriefings. In terms of relevance for their task management, the rating of the debriefings varied considerably. In the following, we discuss several factors that might have influenced characteristics, feasibility and content of the debriefings and might explain the differences between the sites.

In Hospital A TALK was introduced as an extra activity during surgery. In this site, relatively more debriefings were conducted, however, there is the danger of overburdening the clinicians involved [18]. The numerous pauses and utterances as “uhhh” and “what?” and “say that again” that appeared in debriefings during skin closure may be a testament for such overburdening. This overburdening shows three aspects: 1) the need to adapt the debriefing conduct to the current case, 2) learning might decrease, or 3) the debriefing might be terminated. On the other hand, all team members are still in the OR and have the possibility to engage in the debriefing. Completing a surgical procedure can be compared to the “sterile cockpit rule”, where, ideally, no disturbances or interruptions should be allowed [19, 20]. However, in practice, skin closure is sometimes combined with the “Sign out” phase described in the introduction [21]. This “sign-out” is by definition focused on the concrete treatment of the current patient. Combining these aspects with a debriefing that looks beyond the current patient and overarching learning possibilities for the individual, team, and organization could stimulate *team reflexivity* [22, 23] Team reflexivity builds shared mental models as well as triggering team adaptation and learning.

In Hospital B, the debriefings were conducted between cases. There were less signs of overburden and more anesthesiologists could attend the debriefings. In Denmark, they would typically not be present during the operation, where the nurse anesthetist maintains anesthesia. It seems reasonable to presume, that the team gathered between procedures could be more focused on the debriefing, as they do not have to manage the operation in parallel. However, in practice, the team members had often difficulties in finding time to attend the debriefings at this point.

Arguing for an optimal timing of the debriefing is difficult, as there is no “spare time” to use for this extra activity. Placing debriefings at the end or between procedures would need to be an integrated part of completing a procedure to create the time and framework needed, where the respective advantages and disadvantages would need to be balanced.

Regarding external facilitators at Hospital A and internal facilitators at Hospital B, this study does not allow to draw clear conclusions about either approach, as the differences in the relative number TALK debriefings conducted might be due to other factors, as discussed above. Debriefings in both settings were seen equally relevant and resulted in similar content being discussed. Exploring the highest-rated debriefings against the lowest-rated debriefings in both settings indicates a variation in the dialogue. In the highest-rated debriefings, the teams conducted debriefings with less support from the facilitator, and the surgeon typically played an active role. The surgeon is usually considered the leader in the OR and the person who carries the main responsibility for the patient and the surgical procedure [24]. Surgeons also play an important role in facilitating the development of teams and empowering them in their work through their own active participation and effective leadership [25]. That could mean that, in the debriefing context, an active surgeon's role could carry over from the procedure to the debriefing and is likely to engage the rest of the team, which could ultimately reduce the need for a facilitator, especially, if surgeons would get training in the facilitator role. Assumingly it would strengthen a learning and feedback culture in general if OR teams were trained to conduct debriefings themselves and using only internal facilitators would ease practicalities, and with training and insight into the potential benefits of clinical debriefings, also motivation. The surgeons would be important stakeholders in this regard.

Relevance ratings of each debriefing were included to give an indication of the team members’ motivations for the debriefings, which could have an impact on the benefits they gain from debriefings. Motivation and drive, combined with content and interaction, are preconditions for learning [26].

Difficulties in disseminating other checklists in the OR as for example WHO's Safer Surgery and SURPASS [27] have shown barriers that include confusion regarding practical aspects of checklist use, dealing with challenges to efficient workflow, and the beliefs and attitudes of participating staff, particularly surgeons [28]. It is reasonable to assume, that these barriers are also relevant in the current study. The practicalities and dealing with efficient workflow have been discussed above. As for beliefs and attitudes, in this study, the clinicians were asked to rate the debriefings for how relevant they found it for their task management.

In this study, the conversations in the highest-rated debriefings were more lively and louder than those in the lowest-rated debriefings; they typically took place following challenging events, suggesting high motivation in these debriefings. This relates to the traditional view regarding safety, where the understanding of safety derives predominantly from extra-ordinary events, especially looking at failures or mishaps. A new view on safety argues that we should also explore everyday activities that enable successful surgical procedures in order to understand how to apply this knowledge to conscious future practice [29]. These important daily activities enable teams to adapt their behaviors and patient care to the varying conditions of the patient, the team, and the equipment. Everyday activities may not be easy to distinguish and are usually given scant attention and value [30]. Continuous debriefings regarding them may enhance team awareness of success and how to replicate this in a conscious way.

The debriefings were rated very differently in terms of relevance by the individuals involved. It is beyond the scope of this work to describe the reasons for those differences, it would seem interesting to explore, for example, the match between the content of the discussion and the profession and concrete tasks of those involved; the process of the conduct – for example the talking time of those involved, or other influences.

NTS were discussed by all the teams in this study. This may reflect that NTS is what the team members have in common. The teams were encouraged to discuss issues they found most relevant, and they decided themselves what to talk about. “Planning and preparing” and “thinking ahead” was most commonly discussed. It can be seen as the teams attempts to establish shared mental models and increase team reflexivity in the debriefings.

The ethos of the TALK tool is that teams themselves decide when to debrief and that anyone can initiate a TALK if he or she feels a need for it and is familiar with the tool [11]. In this study, the teams were asked to debrief after defined procedures in day shift, whether they found it relevant or not. The intention was to establish a debriefing habit among staff and to make them practice, to make it easier for them to conduct an effective debriefing when a need actually exists. The project gave the staff a possibility to try the tool and see if it provided added value.

Another aspect that might enhance motivation for clinicians to conduct debriefings, could be the importance of feedback from the organization on how insights derived from the debriefings has been handled. It would be demotivating to discuss same problem or issue in one debriefing after another without knowing whether the organization deal with it. Outcome-driven clinicians will likely be motivated by recognizing that their ideas do have an impact. This feedback loop would be an interesting aspect to investigate in future studies.

Implementing a team communication tool in a complex, production-oriented work environment, where no time is allocated to team development systematically, is a challenge which presumably will influence the possibility for teams to learn from each other and the situations they have been through together.

There are likely a range of other factors influencing, numbers of debriefings, feasibility and content. An investigation of those is beyond the scope of this project. Yet, the difference in how many debriefings were conducted emphasizes the need to consider the implementation of debriefings in clinical contexts.

### 4.1 Study limitations

The study design balanced requirements of a clear study design with the actual possibilities in the sites investigated. We could collect relevant data in the clinical environment, consequently introducing some challenges to the possibility to compare sites with each other. Even so, exploring feasibility in each Hospital has provided interesting information on advantages and disadvantages of each way of practicing debriefings in the OR and underlined the need to emphasize the concrete implementation of a tool like TALK. Our study has several limitations that we discuss in the following.

One risk of bias could be related to having external facilitators present in some of the debriefings, because participants might be less open in the discussions. Also, audio-recordings of the debriefings might have had similar effect. Nevertheless, serious topics and mishaps were discussed several times in a constructive manner. One could also speculate whether external facilitators made it possible for the teams to conduct the debriefings at all, as they might have given the impulse to do so.

In relation to the relevance ratings, several team members expressed confusion about how they should interpret the question: whether they found it relevant personally or relevant to the patient, whether the surgery was performed well, or whether they thought, the issues discussed would improve future patient care. This uncertainty could have affected the ratings and contributed to the range in the ratings. Moreover, the ratings were performed in a way that other team members could hear them. This might have led to anchoring effects, where the first pronounced rating might have influenced the following ratings by other team members. Even so, there were debriefings where ratings within the teams differed. Lastly, the departments were selected based on their track records of working with patient-safety projects, we assume that this likely leads to a more positive picture about the debriefings as compared to average departments.

### 4.2 Implications for clinical practice

Different clinical implications can be drawn from the current study. During the study, team members decided to omit debriefings after short procedures, as they experienced a mismatch between the duration of the debriefing and the procedure. A way to increase the perceived value of the debriefing might lie in collaboratively developing trigger criteria for a debriefing with those involved. This could be events (e.g. blood loss above 250 ml) or specific procedures (e.g. any surgery exceeding two hours or any laparoscopic colon resections). The latter approach might trigger insights that are beyond right and wrong actions. The conduct might benefit from jointly deciding, what would trigger the conduct of the debriefing and discussing relevant points in time, as team members performing concurrent task might miss the “window of opportunity” for a debriefing. Recognizing the surgeon as an important team member in the highest-rated debriefings seems to emphasize his or her impact on the debriefing's perceived relevance and potential to trigger its conduct.

### 4.3 Future studies

Future studies could investigate how much training is needed for clinicians to be able to conduct debriefings themselves without external facilitators and what other factors influence the motivation and ability to engage in this type of conversation. Furthermore, studies could include research on how organizations can learn from clinicians and vice versa, specifically, how gained insights and intended actions discussed in the debriefing are handled personally, in the team and in the organization. Finally, assessing the effects of debriefings on surgical time, blood loss, time between procedures, and/or the patient-safety culture could be valuable.

## 5 Conclusion

In conclusion, feasibility was challenged at both sites, presumably due to the timing of the debriefing, experience of the facilitators, and team composition. Nevertheless, the teams succeeded to reflect on practice and discussed non-technical skills in all the debriefings in their production-focused, complex work environment. Predominantly, the team members rated debriefings as relevant following challenging events and when surgeons actively participated in the conversation. A range of concrete suggestions on how to improve practice resulted from the debriefings.

## Declarations

### Author contribution statement

A. Mundt: Conceived and designed the experiments; Performed the experiments; Analyzed and interpreted the data; Contributed reagents, materials, analysis tools or data; Wrote the paper.

K. Gjeraa, L. Spanager, and S. Petersen: Performed the experiments; Contributed reagents, materials, analysis tools or data; Wrote the paper.

P. Dieckmann and D. Østergaard: Conceived and designed the experiments; Analyzed and interpreted the data; Contributed reagents, materials, analysis tools or data; Wrote the paper.

### Funding statement

This research did not receive any specific grant from funding agencies in the public, commercial, or not-for-profit sectors.

### Competing interest statement

The authors declare no conflict of interest.

### Additional information

No additional information is available for this paper.
